# Directly engaging patients with glucose management: integrated visualization of personal device data for use between traditional visits

**DOI:** 10.1016/j.hfh.2026.100131

**Published:** 2026-03-31

**Authors:** Emily S. Patterson, Joshua E. Lacko, Erin Der-McLeod, Vanessa I. Chuang, Garrett I. Ash, Constance H. Fung, Selene S. Mak

**Affiliations:** a School of Health and Rehabilitation Sciences, College of Medicine, The Ohio State University, Columbus, OH, USA; b VA Center for the Study of Healthcare Implementation, Innovation and Policy (CSHIIP), VA Greater Los Angeles Healthcare System, Los Angeles, CA, USA; c VA Geriatric Research, Education and Clinical Center, VA Greater Los Angeles Healthcare System, Los Angeles, CA, USA; d Department of Medicine, David Geffen School of Medicine at the University of California, Los Angeles, California, USA; e Department of Internal Medicine, Section of General Internal Medicine, Yale School of Medicine, New Haven, CT, USA; f Pain, Research, Informatics, Medical Comorbidities and Education Center, Vetrans Affairs Connecticut Healthcare System, West Haven, CT, USA

**Keywords:** Consumer informatics, Shared decision making, Visualization design

## Abstract

Keeping glucose below a threshold, as well as minimizing variation, is critical for individuals living with type 2 diabetes to avoid harm to organs and increase length and quality of life. Wearable devices provide patients with the opportunity to gain insight into how their physical activity and sleep quality relate to glucose levels monitored on a continuous basis. With integrated displays combining data from multiple wearable devices as well as self-reported data and assessment, there is an opportunity to see how related choices interact together to affect glucose levels on a continuous basis and averaged over the day, including documenting more information about diet, sleep, mood, and other activity choices. We conducted interviews with patients to assess their understanding of and anticipated strategies to use a personalized integrated display in-between traditional visits without the synchronous support of a clinician. We aimed to: 1) Reveal what information and uses for an integrated report are valuable for patients and 2) Elicit formative suggestions for improvement of the design of the report. An interview protocol was employed to investigate the reactions of patients to an integrated report (*N* = 11). The results underscore that integrated visualizations primarily support patients in making sense of their own data obtained from multiple, independent wearable devices. With an integrated report, they can link their health behaviors to glucose variation, motivating self-reflection and behavioral change, and enhancing self-efficacy. Further, participants valued access to their own data and made recommendations to improve the usability of diet and mood information display. We discuss how integrated visualizations can enhance supervisory control and shared decision-making by empowering patients to act as informed partners in shared decision making with clinicians and diabetes self-management between visits.

## Introduction

Over the past decade, the clinical time in the traditional office visit in primary care has gotten shorter, (Tai-Seale, McGuire & Zhang, 2007) with less financial compensation for primary care providers, (Holmgren, Adler-Milstein & Apathy, 2024) increased expectations from additional guideline-based care, (Porter, Boyd, Skandari & Laiteerapong, 2023) and required higher documentation expectations. (Holmgren, Adler-Milstein & Apathy, 2024) Over the same period, there has been an increase in the scope of care provided by nurse practitioners and physician assistants instead of medical doctors in primary care (Waxman & Dechene, 2024) and increased use of health and wellness coaching (Veterans Health Administration, 2025) to complement primary care, often between traditional visits. (Gastala, Wingrove, Gaglioti & Liaw, 2018) Health and wellness coaching can increase behaviors such as exercise, which can reduce cardiovascular risk by increasing aerobic capacity, lipid management, and glycemic control for patients with diabetes. (Wu, Bredin & Guan, 2019) From a human factors perspective, we need to design a new coordination paradigm between traditional medical doctors or less-trained primary care providers and health and wellness coaches (hereafter “coach”) to coordinate care for patients living with type 2 diabetes (T2D) and increase patient self-efficacy for self-management.

For patients with T2D treated in primary care, keeping glucose below a threshold, as well as minimizing variation (i.e., “in range”), is critical to avoid harm to organs and increase length and quality of life. Lifestyle modifications and emerging medications are two of the most common treatments. (Tegegne, Adugna & Yenet, 2024) Lifestyle modifications, supported by a coach and the use of patient-generated health data (PGHD) from wearable devices such as smartwatches, continuous glucose monitors, and fitness trackers, are a promising area for care innovation that can be done outside of a traditional office visit in primary care. While these data streams offer unprecedented opportunities to engage patients in their own care supported by coaches, they often remain “theoretically available,” meaning patients theoretically have access to the data but in disparate form, and the data are often challenging to interpret without assistance. Inspired by the literature on supervisory control in complex, semi-automated systems (e.g., aviation and process control),9 we explore how to design useful integrated visualizations of how lifestyle modifications tracked by wearable devices relate to blood glucose, using data from continuous glucose monitors (CGMs) and other digital health tools (e.g., smartwatches, mobile applications). Supervisory control models emphasize the role of a human operator in interpreting feedback from automated systems, detecting emerging problems, and adjusting actions. (Sheridan, 1992; Parasuraman, Sheridan & Wickens, 2000) Related human factors work on level of control and operator performance in automated environments further motivates attention to how data are organized and interpreted by users. (Endsley & Kiris, 1995) This perspective conceptually aligns with diabetes self-management supported by integrated PGHD displays. (Parasuraman, Sheridan & Wickens, 2000) In this framing, patients function in a supervisory control role for glycemic self-management, while coaches and clinicians support interpretation, planning, and escalation when needed.

Whereas supervisory control and macrocognition frameworks describe how individuals interpret and adapt to dynamic information environments, the IMB model specifies the conditions under which such information processing translates into sustained health behavior change. In this project, we combine theoretical concepts from the human factors literature with the Information-Motivation-Behavioral Skills (IMB) approach that underlies health coaching interventions. IMB identifies three conditions for individuals to adopt and maintain health behaviors: 1) relevant information about what needs to be done, 2) motivation to act, which includes both personal and social elements, and 3) behavioral skills, which include self-efficacy and the capability of executing the desired health behavior. (Fisher, Kohut, Schachner & Stenger, 2011) In our prior research encouraging exercise for persons with type 1 diabetes, relevant information was provided from CGM data, as well as sleep and activity patterns from smartwatches. Motivation was enhanced with individually tailored discussions that included goal setting. Behavioral skills were supported regarding timing exercise activities and adjusting insulin dosing based on activity. (Ash, Nally & Stults-Kolehmainen, 2023)

In naturalistic decision making, a subset of human factors, macrocognition functions (Patterson & Hoffman, 2012) have been identified at the unit of a Joint Cognitive System (JCS), meaning that cognition functions in a distributed manner across experts in specialized roles and supported by sophisticated artifacts. (Roth, Patterson & Mumaw, 2002) These artifacts can include embedded automation that acts in ways that share some commonalities with human agents, which also needs to be coordinated to achieve a particular macrocognition function. (Li & Burns, 2017) When providing care for patients living with T2D, the five macrocognition functions roughly translate to diagnosis (a.k.a., sensemaking), treatment planning (a.k.a., re-planning), detecting when problematic events occur with patients (a.k.a., detecting problems), reassessing commitment to prior decisions made for future actions (deciding), and coordinating care, including with the patient (coordinating).

From the human factors literature, a relevant concept is how to tailor coordination architectures for collaborative activity based upon levels of expertise and experience. For example, the ‘generalist-specialist problem’ (Woods, 1985) describes challenges that suggest the need to support experts with broad, yet limited depth, expertise to identify when to recruit specialists with deep expertise in relatively small knowledge domains during sensemaking and (re)planning. (Ericsson, Hoffman, Kozbelt & Williams, 2018) Coaches help patients make sense of the data in between traditional clinic visits so patients can apply behavior insights. Patients have been identified as knowledge workers with deep knowledge about their own bodies and medical histories. (Papautsky & Patterson, 2021) Therefore, patients can be supported in identifying when to obtain support from coaches, primary care providers, and diabetes specialists. Particularly important skills are identifying what information to share in what scenarios and what questions to ask at the next scheduled visit.

Building on the supervisory control framing introduced above, the increased sophistication and availability of CGMs and wearables, such as smartwatches, provide new data directly to consumers that were not previously available to either consumers or health professionals. With these data, patients can directly obtain and act on information to change their health behaviors without involving a health professional. Seeing how behaviors impact glycemic control can provide motivation to continue to improve control. We are interested in how to build upon this new capability by supporting shared views of currently disparate data sources for patients, family members, coaches, primary care providers, and endocrinologists. Our design efforts for an integrated display of inputs from wearable devices and self-reported dietary, mood, and sleep data are influenced by human factors insights on how to reveal relevant complexity with temporal-based displays showing interactions among input and output variables.

In this study, the IMB framework and macrocognition functions provide complementary lenses for understanding how integrated PGHD visualizations may influence patient self-management. IMB emphasizes the conditions necessary for behavior change (information, motivation, and behavioral skills), while macrocognition functions describe how these elements can be extracted from the complex 21st century diabetes technology environment.

Guided by these frameworks, we conducted a project informed by the IMB framework, coordination architectures for collaborative care with practitioners of varied expertise and experience, and supervisory control as a lens on a patient’s glycemic self-management. With this project, we aimed to 1) reveal what information and uses for an integrated report are valuable for individuals with diabetes supported by coaches between clinic visits and 2) elicit formative suggestions for improvement to the design of the report.

## Project questions

The following project questions were developed to align with IMB theory and macrocognition functions:

1. Do patients anticipate using the integrated reports across care contexts (e.g., during traditional visits versus in-between visits), and how do these contexts shape macrocognitive coordination and supervisory control in diabetes self-management?
2. How do patients anticipate obtaining clinical support when using integrated reports, and how do they envision macrocognitively coordinating actions or replanning behaviors across asynchronous and synchronous care interactions?
3. What information is needed in integrated reports to support motivation and behavioral skills for health-related behavior change?
4. What macrocognitive functions (e.g., sensemaking, replanning, detecting problems) do patients perceive the integrated reports as supporting?
5. How could the design of the reports be improved?

## Method

### Participants

Eleven patients currently being treated at the Veterans Health Administration (8 men and 3 women) comprised a sample of patients with an age range from 37 years to 78 years old living with a diagnosis of T2D. Ten were on multiple daily injections of insulin and one was on an automated delivery pump. Inclusion criteria for participation were receiving care in the Connecticut Veterans Administration Medical Center and using a CGM and no health conditions that would preclude lifestyle conditions with a coach, such as psychiatric hospitalizations or living on dialysis. A $80 gift card and a smartwatch were offered to participants for participation.

### Materials

A project staff member conducted semi-structured interviews using an interview guide. The interviews were all conducted remotely; they were digitally audio-recorded and automatically transcribed using Microsoft Teams. The first two participants were shown a report with an integrated visualization on one page of data from the patient’s device and self-reported data, consistent with typical user-centered cognitive interview procedures. However, the highly individualized nature of these reports, particularly with respect to self-reported mood and dietary data, introduced substantial variability in the interview stimulus. To reduce this variability and improve comparability across interviews, the remaining nine participants received a report generated from a simulated fictional patient’s data.

**Fig. 1.**
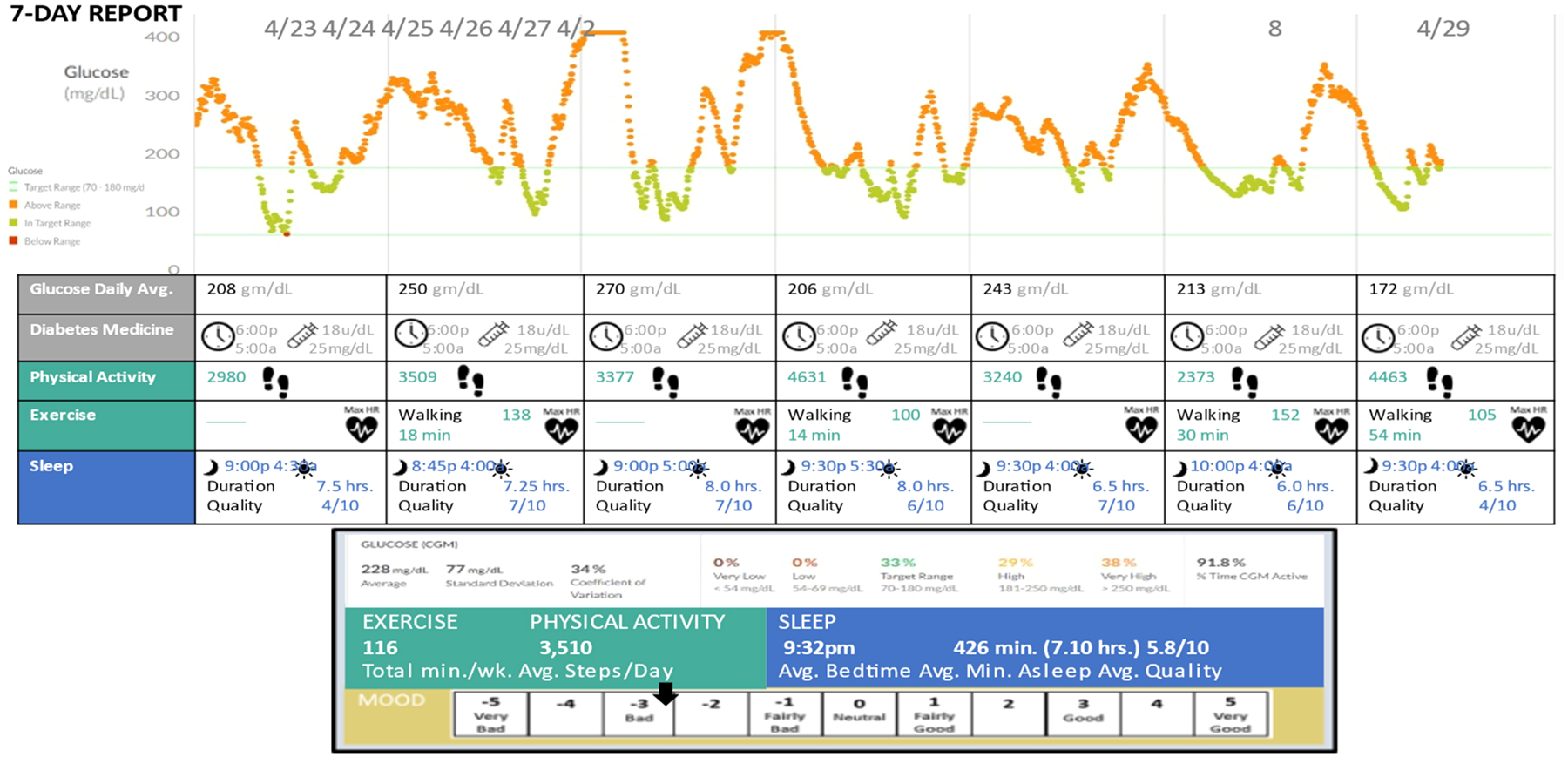
Integrated 7-Day Report for a fictional patient provided during interviews with nine participants.

An example of a simulated fictional patient’s 7-day integrated report is provided in Fig. 1. At the top of the report is the glucose level from the CGM, with daily averages summarized directly below. Information drawn from smart devices worn by the patient includes the number of steps taken each day as a measure of physical activity, and also included are data about exercise minutes and maximum heart rate while exercising. Self-reported sleep data are displayed at the bottom each day. Self-reported mood information over a seven-day period is displayed on the bottom of the integrated summary at the bottom of the report.

**Fig. 2.**
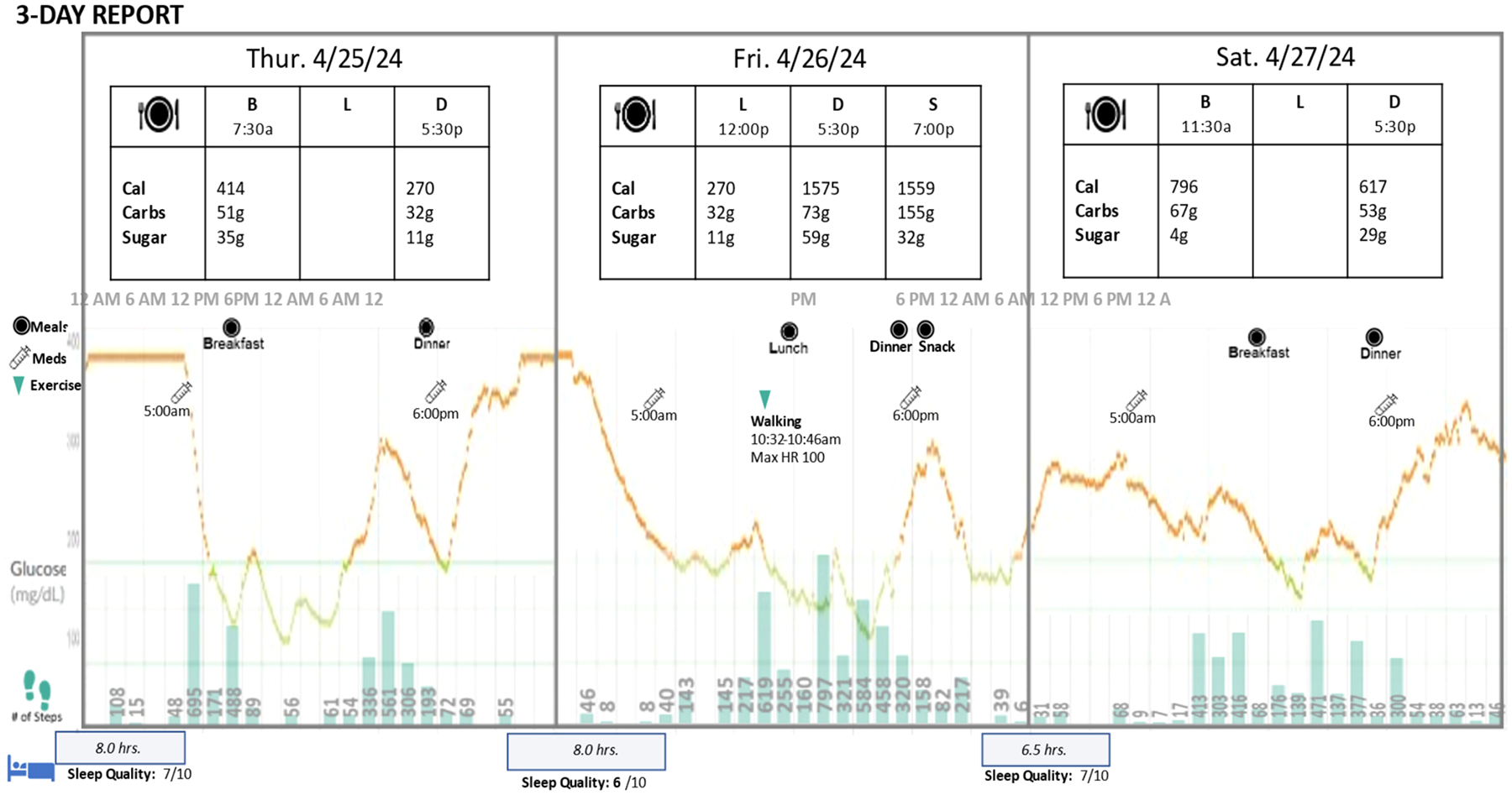
Integrated 3-Day report for a fictional patient provided during interviews with nine participants.

An example of a simulated fictional patient’s 3-day integrated report is provided in Fig. 2. Compared to the 7-day report, additional dietary information is provided, with a summary of calories, carbohydrates (in grams), and sugar (in grams) for breakfast, lunch, snack(s) and dinner. The patient self-reported what foods were eaten, and this summary transforms that information into these categories. In addition, the timing of breakfast, lunch, dinner, and snacks are added to the timeline display, as is the timing and type of exercise and administrations of diabetes medication. The number of steps taken per hour is added to the bottom of the graph. Finally, the duration and quality of sleep is added for each day.

### Procedure

#### Interview questions for 3- and 7-Day report

1. In the 7-day Report:
  - Which part did you start with—the upper left? Right? Bottom?
  - What do you make of the numbers and symbols displayed within the chart?
  - How did you arrive at that answer? Was it easy or hard to answer? [I noticed that you hesitated –tell me what you were thinking] [Tell me more about that]
  - For the table section below the 7-day report, what do you think is meant by the total values shown in this section? How are these values related to the report above?
2. In the 3-day Report:
  - Which part did you start with—the upper left? Right? Bottom?
  - What do you make of the numbers and symbols displayed within the chart?
  - How did you arrive at that answer? Was it easy or hard to answer? [I noticed that you hesitated –tell me what you were thinking] [Tell me more about that]
3. Additional questions as time allows:
  - What was your first impression (general thoughts) of the report? Is it inviting, professional, biased? Was there any difficulty in reading it? What’s your opinion on the design, formatting, color scheme, length?
  - Is there any part that you need clarification on? (e.g., repeat instructions needed, clarification)
  - Is there anything that should be added to the report? (Are there any areas where it’s lacking information? What areas? What should be added? What other information would you like to see?)
    - Probe: What concerns with the report do you think other patients would have that we have not covered?
4. Is there anything that we should remove from the report? (Anything that felt redundant or unnecessary?)

### Design and analysis

Interview notes and transcripts were analyzed using structured content analysis with predefined coding categories. Comments were segmented into discrete units and categorized by PGHD domains aligned with report sections (e.g., glucose, activity/steps, sleep, diet, medication, mood), macrocognitive functions reflected in participants’ described uses (e.g., sensemaking, problem detection, deciding/replanning, learning), and IMB domains (e.g., information, motivation, behavioral skills). Five team members contributed to coding. One investigator performed the initial segmentation of comments into discrete units, after which each unit was independently coded for comment type (positive valence, negative valence, or suggestion) by two reviewers from the five-member team; reviewer pairings varied across units. Statements that did not clearly align with predefined categories were coded as not applicable. Discrepancies were resolved through discussion to create the final coded dataset. This approach was applied consistently across analyses reported in Tables 1–4.

Table 1 summarizes the coding structure used to analyze transcript data according to the IMB framework. Statements were classified as reflecting information (gaining insight or understanding), motivation (increasing readiness or confidence to act), or behavioral skills (planning or observing a change in behavior or outcomes). Statements that could not be clearly assigned to one of these categories were coded as *not applicable*.

In Table 2, the same transcript data analyzed in Table 1 were further analyzed with respect to the elements included in the reports, including sleep, exercise, diet, medicine (e.g., insulin), mood, or controlling glucose levels in general.

For macrocognition coding, transcript excerpts were reviewed to identify functional processes supported by the report, and each excerpt was assigned to a single dominant macrocognitive function through consensus among investigators. In Table 3, the transcript data were coded for which macrocognition functions the integrated report might support. In this case, macrocognition occurs in a distributed manner across the primary care provider, health coach, patient, patients’ family members, automatic data capture by wearable devices, provision of information in the electronic health record, and automated, manual, or hybrid construction of the integrated report. Many of the transcribed comments were further parsed to enable unique categorization of a macrocognition function. There were no examples of coordinating in the dataset, which likely suggests that the interview questions probed the usefulness of the report as viewed solely from a patient’s perspective.

In Table 4, we provide the codebook examples for the assessment of valence regarding comments on the design and use of the 1) overall design of all integrated reports, 2) design of the 7-day integrated report, 3) design of the 3-day integrated report with additional details regarding diet, exercise, and sleep. The suggestion category provides a recommendation for improvement without using terms that indicate a clear valence; suggestions include strategies to address issues as well as opportunities to build upon positive aspects.

Finally, in Table 5, the steps involved in identifying how the report is useful are provided along with examples. First, the transcript of the interview was parsed to identify chunks for coding. Second, the content of each parsed element was restated by the first investigator [EP]. Then another investigator independently did the same thing [SM]. The two investigators then reconciled their independent descriptions into a single reconciled statement, which included ensuring that there was full consensus on the final description. Then the first investigator categorized the parsed data as a function in macrocognition along with a descriptive sub-theme. Then a meeting was held with three investigators [EP, SM, GA] in which a single reconciled statement was generated with full consensus for each parsed element of the transcripts. Finally, the resulting reconciled statements were grouped and a label was provided by the first investigator for each group of statements.

## Results

In Table 6, regarding the IBMS approach, the intention of the report was to visualize the data in a way that becomes information that enhances motivation for health behaviors. The participants articulated how the information was provided to them in reports, and that the format for providing an integrated view of information was valuable. Regarding motivation, seeing information was enough to motivate participants to change behaviors, including having to self-report what was eaten for meals and snacks motivating improvements in dietary choices. The insights from viewing the report provided actionable insight for behavioral change in some cases. For example, a participant identified that taking insulin while eating resulted in low blood glucose and so would not do those activities together in the future.

Regarding the PGHD domain for the displayed information, most comments regarded controlling the glucose level, which is to be expected, as it is the most prominent feature and impacted by the other inputs. There was approximately equal emphasis on diet, exercise, insulin (medication), and sleep, which were the next areas of emphasis on the report. There were few comments regarding mood, which was at the bottom of the 7-day report and was only reported once with a 7-day lookback period.

Regarding macrocognition, sensemaking was the primary function of the integrated report. This included understanding an overview snapshot at one glance, understanding the impact of changes to behaviors on glucose values and relationships between variables, including the timing of the administration of diabetes medication and glucose values, learning categories of possible actions and thresholds within variables, and getting access to data to aid with making sense of patterns. The report also supported re-planning, including identifying ways to modify behavior to reduce glucose levels and change reporting behaviors to reduce gaps in self-reported data. The report supported detecting problems, including detecting undesirable changes in glucose levels due to eating. The report supported deciding what previously determined future actions to remain committed to or modify. There were no instances of supporting coordinating, which is not surprising given that the integrated report did not explicitly intend to support coordination.

In Table 7, most comments were positive and about the report overall. Most comments on the iconography, layout, design, and color coding were positive. Most negative comments and suggestions related to data collection through self-report and surveys that were needed to display diet and mood data. The tips section had two suggestions for improvement, with no positive or negative comments. Seven positive comments, three negative comments, and five suggestions could not be categorized.

In Table 8. we detail the areas of the 7-day integrated reports where participants provided comments that were categorized as positive, negative and constructive suggestions for improvement. The CGM graph had two positive comments and one negative comment. Valence was mixed regarding physical activity and sleep, with some comments indicating that daily physical activity and daily sleep data might be missed. No positive comments, four negative comments, and five suggestions regarded mood, which was assessed once a day through a survey and displayed below all other information.

**Fig. 3.**
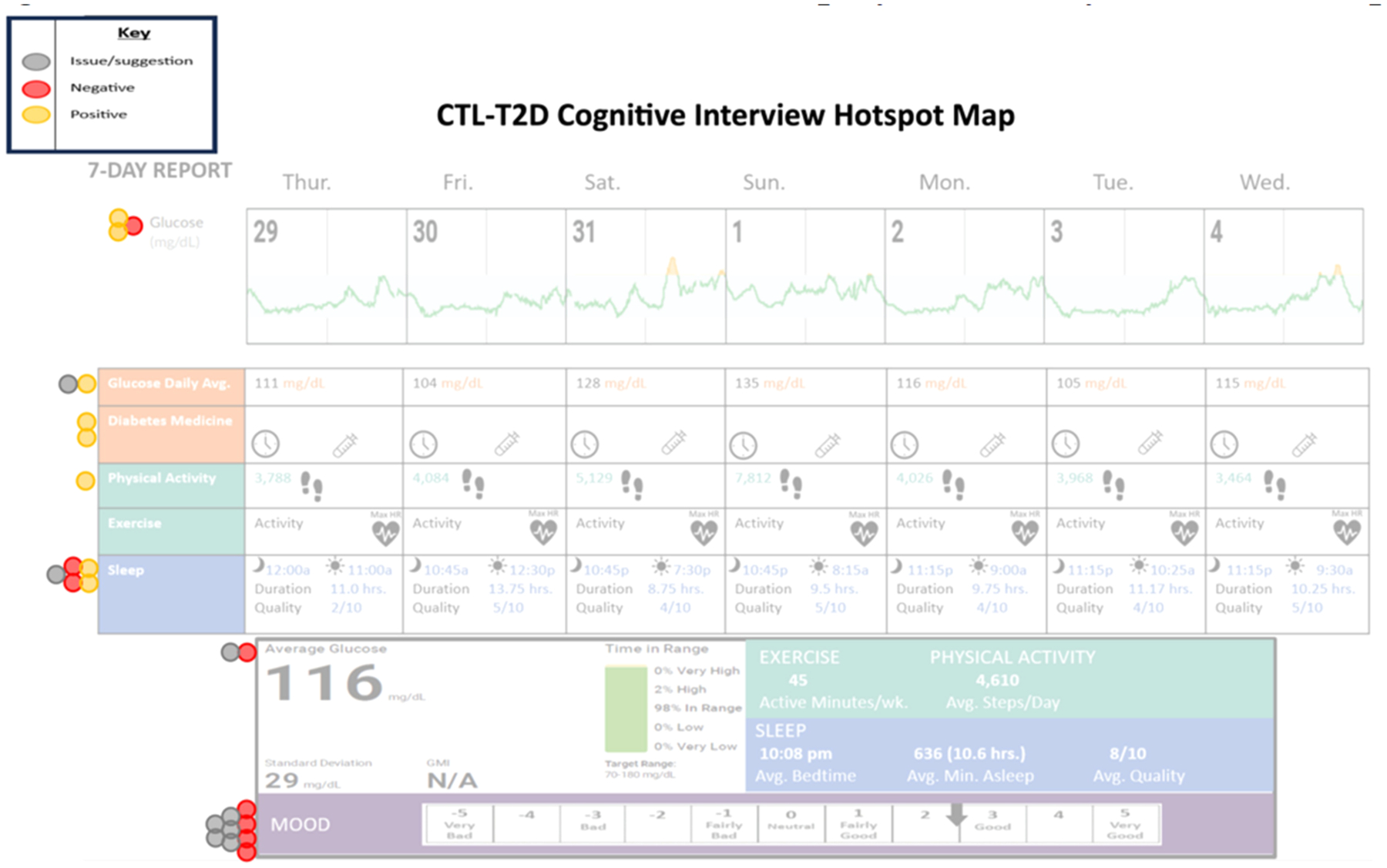
Location of positive comments (yellow), negative comments (red) and suggestions (gray) for 7-day integrated report.

In Fig. 3, the same data from Table 8 are displayed visually as a ‘heat map.’

In Table 9, we detail the areas of the 3-day integrated reports where participants provided comments that were categorized as positive, negative and constructive suggestions for improvement. The sleep plot bar graph was an area of focus for comments, with both positive and negative comments and two suggestions. All overlays in the 3-day integrated report had room for improvement, based upon the negative comments and suggestions.

**Fig. 4.**
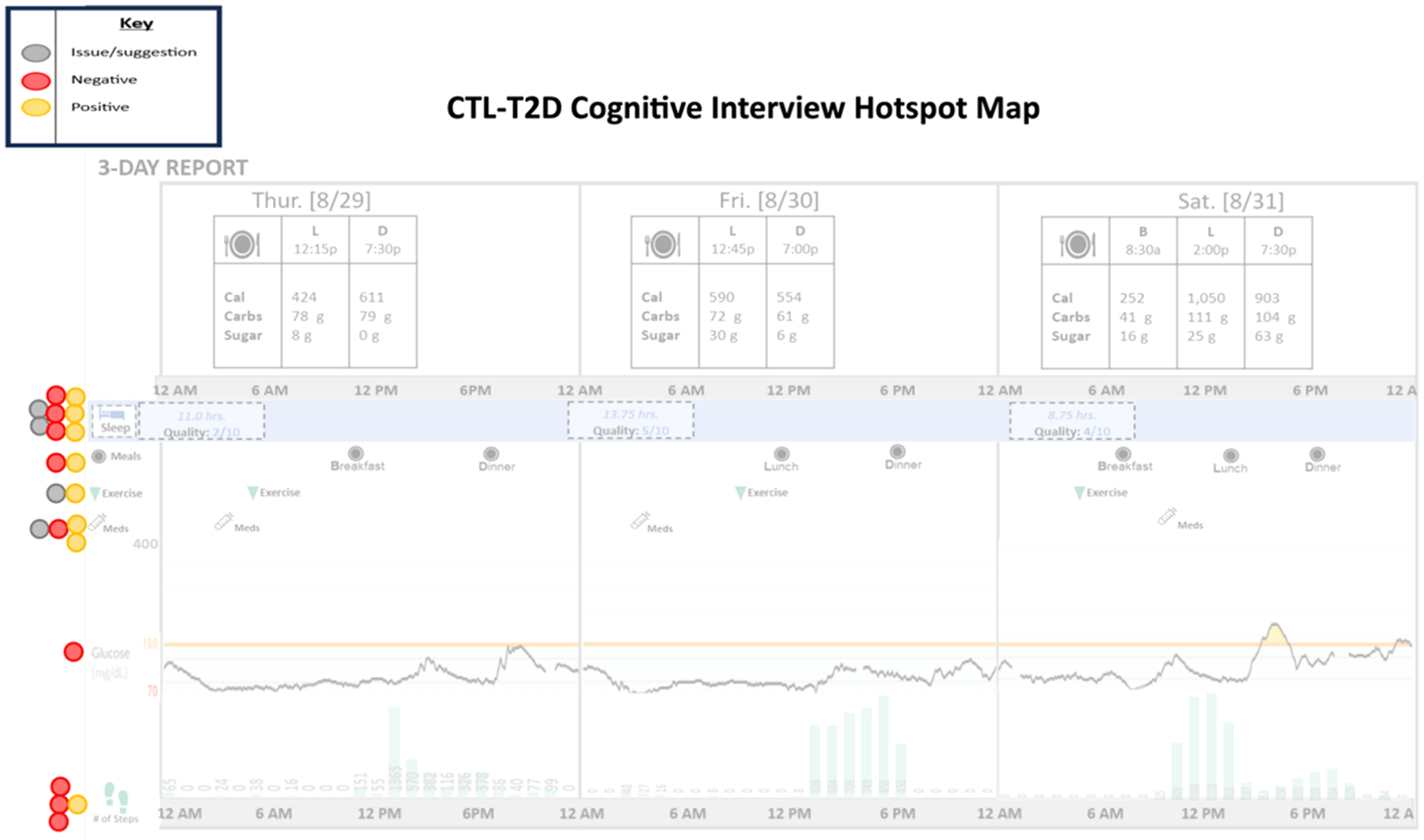
Location of positive comments (yellow), negative comments (red) and suggestions (gray) for 3-day integrated report.

In Fig. 4, the same data from Table 9 are displayed visually as a ‘heat map.’ The positive and negative comments are spread relatively evenly on the 3-day report, although there were somewhat more negative comments in the area with the number of steps displayed per hour.

In Table 10, we present the findings about how the project participants felt that the integrated report would provide value to patients in an actual clinical setting. We identified 11 categories for why the integrated report could be useful for patients. Based on frequency, the most common explanations were 1) providing access to information directly to patients, 2) allowing patients to answer questions directly, with the help of someone to interpret the report, or to ask questions of their primary care physician, and 3) understanding how behavior impacts glucose levels. Four of eleven participants highlighted the importance of showing them their own data in detail, which they would otherwise not have access to. Three appreciated better understanding how their choices impacted their glucose levels. Three felt that the report supported finding patterns. Three participants valued having an overview snapshot in one display. Three participants felt that the report supported asking better questions of themselves and clinicians. Two participants appreciated the graph format for supporting this. Two felt that a clinician would need to interpret the report for them. All comments were either stated in a neutral or positive valence. Some were stated as conditions for usefulness, such as requiring clinical knowledge. Some were stated as suggestions for how to make the report even more useful, such as adding ability to get more details upon clicking an interactive version of the report.

## Discussion

We explored how patients with T2D interpreted and anticipated using integrated reports that combine data from CGM, smartwatch, and self-reported inputs such as diet, sleep, and mood. The interviews revealed that participants primarily used the reports for sensemaking, which support linking specific behaviors to variations in glucose levels and identifying patterns across time. The 7-day and 3-day versions supported different levels of insight in that the 7-day summary allowed an overview of patterns, whereas the 3-day detailed display enabled fine-grained examination of relationships among behaviors, diabetes medication timing, and glucose variation. Patients described how these visualizations provided actionable information, motivated them to adjust behaviors, and facilitated planning for future actions.

The integrated visualizations thus served as a bridge between information, motivation, and behavioral skills within the IMB framework. Information emerged as the foundation in that participants valued access to their own data in a coherent, comprehensible format. The behavioral implications of this information distinguished this tool from others that merely provide numerical feedback. Viewing multiple physiological data streams together transformed raw data into insight, supporting patients’ motivation to make meaningful behavior changes.

From a human factors perspective, the findings illustrate the principle of supervisory control applied to diabetes self-management. Patients function as supervisory controllers who monitor complex, semi-automated systems, in this case, their own glycemic regulation informed by wearable and self-reported data. The integrated reports supported supervisory control by reducing complexity and providing different levels of abstraction. The 7-day report afforded a higher-level overview for recognizing trends and maintaining situational awareness, while the 3-day version enabled detailed inspection of relationships among diet, sleep, activity, medication, and glucose. This layering of information aligns with established display design principles, allowing users to move fluidly between overview and detail, thus improving cognitive manageability in the face of abundant personal data.

The inclusion of overlays, particularly in the 3-day report, was novel for participants and elicited some negative comments that indicate the need for orientation and training. Most had not seen their health data integrated in this way. While this complexity required some learning, it provided a richer, more explanatory model of how everyday choices influenced glucose control. Such visual integration supports building a shared understanding between patients and their coach regarding interactions between health behaviors and their impact on continuously monitored glucose levels.

Participants expressed that self-reported data, while burdensome to input, gained new meaning when visualized alongside continuous and automatically captured sensor data. The visualization made the effort of self-reporting worthwhile in that it contextualized states obtained through logs and surveys (e.g., mood, meal and snack content) within the objective patterns of glucose and activity. The process also reinforced accountability and reflection. A few participants’ comments about the mood display illustrate the challenge of balancing comprehensiveness and interpretability. Participants found weekly mood entries less informative, prompting a redesign that replaced numeric scales with icon-based representations and shifted mood data into summary sections. Future iterations may benefit from insights from ecological interface design. (Burns & Hajdukiewicz, 2004)

These findings also align with the broader specialist–generalist challenge (Woods, 1985) in that patients act as generalists with broad, experiential knowledge of their bodies but limited depth in medical knowledge. Clinicians, conversely, are specialists with deep knowledge of their area of medicine, but limited visibility into the patient’s lived experience. The integrated reports help bridge this gap by equipping patients with tools to recognize when specialist input is needed and what questions to ask of clinicians between and during visits. By identifying patterns or anomalies between visits, patients can bring specific, data-informed questions to clinical encounters, supporting moving from passive recipients of care to active collaborators.

Extending this concept, patients can also be viewed as knowledge worker, (Papautsky & Patterson, 2021) or individuals with unique expertise in their own physiological and behavioral data. Effective care requires clinicians to know when to access patients as “specialists” on their own histories and bodies. The integrated report serves as a boundary object that supports this bidirectional expertise exchange in that it gives patients the means to synthesize and communicate insights and gives clinicians a structured artifact to interpret PGHD efficiently. This design therefore promotes shared decision-making; there is a redistribution of cognitive work where patients contribute interpretive insight about patterns found in the data as well as self-report data at the time that it is best to elicit it that would not otherwise be available to the clinician.

### Limitations and future directions

This project had a number of limitations. There was a relatively small number of patients who participated in the project, and there is a wide range of variability in symptoms experienced with T2D and willingness to adopt new technologies. One of the participants had an automated delivery pump, which may have influenced their experience substantially compared to the other participants’ experience with T2D management. One investigator reviewed all data from that individual to see how the findings would be impacted if their data were removed, and no differences were found. Similarly, the insights from the first two participants were qualitatively compared against the other nine and insights were generated on the same content categories. There were some differences in that some insights from the first two participants were generated from remembering explanations for changes in behavior captured in the report, such as attending a party, while the other nine participants had reviewed a simulated report.

## Discussion

In summary, our findings suggest that our approach is promising for supporting patients with T2D:

1. Presents information at multiple levels of abstraction (overview and detail).
2. Reduces cognitive load by integrating related behavioral and physiological data.
3. Provides meaningful feedback loops that justify the effort of self-reporting.
4. Enables patients to act as informed partners (knowledge workers) in collaborative care.

## Figures and Tables

**Table 1:** Codebook with categories and examples for the integrated report relating to the IMB approach.

Category	Example
**Information**: Get information and insight	Yeah, my blood sugar is gone way down after breakfast and it’s kind of hanging around down there in that sort of zone and then it begins to creep up again.
**Motivation**: Increase motivation to improve health	I feel I eat a lot less when I have to log it… Yeah, it was the same way when I was doing the Weight Watchers.
**Behavior**: Plan to make a change, or see impact of a change in behavior	I think I need more sleep number one, but when I'm sleeping, the blood glucose level, 1st of all stay within a target range and except that Monday sure.

**Table 2:** Codebook with categories and examples for the integrated report relating to the clinical data in the integrated report.

Category	Example
**Control**: Glucose level and relation to thresholds	I'm what I'm thinking is like I said before, there's a lot of information here and I think it would be very helpful if you had some conclusions. But slide with a little bit of conclusion, saying you're not getting enough sleep. You're not getting, you're not walking enough. You're eating too much of this. Uh. Maybe cut back on sugar. You know, just a little bit of summary of some kind of conclusion, so that they don't have to make all the conclusions themself.
**Diet**: Timing of meals, calories, carbohydrates, and sugar	I know I noticed that on Friday. His did not do breakfast, but his lunch, which we cannot compare with from the day before because he didn't do lunch the day before. So there's nothing to compare it with, but it's a dynamical to what he did the night before for dinner.
**Exercise**: Activity, timing and number of steps	OK, while the physical activity is the steps obviously with the footprints there, that's pretty easy exercise it probably it. It tells you when you're walking on this particular chart, it may at some point show running or some other type of exercise, I imagine, but it tells you how many minutes you were.
**Medicine**: Administration of insulin	So this 18 units and 25 Milligrams. I don't understand what 18 units is. Probably insulin. The 25 mg, I don't know what that is.
**Sleep**: Duration and quality	Because everything that you're reflecting reflects how we are as diabetics. If you don't get enough sleep, your sugars are gonna be off. If you get too much sleep, your sugars are gonna be off. If you don't exercise enough, your sugars are going to be off. Everything plays a big part in our shares.
**Mood**: −5 (very bad) to 5 (very good)	And then mood, bad mood, hmm? So the mood? Now, where does that come? Where does that come from? Is that from the survey also?

**Table 3:** Codebook with categories and examples for the integrated report relating to macrocognition functions.

Category	Example
**Sensemaking**: Includes activities of collecting, corroborating, and integrating information and assessing how the information maps onto potential scenarios or explanations	it's something that you can read. It's, it's a lot easier to understand and I don't have to try to remember what I ate this day, why is this going up that day? Did I take the medicine? It, everything's laid out for me.
***Re*-planning**: Adaptively responding to changes in objectives, from any of a variety of sources including supervisors and peers, obstacles, opportunities, events, or changes in predicted future trajectories	OK, I have to wait until my blood sugar starts climbing before I take insulin. Mm-hmm. OK. You know, and that's where I love the constant glucose monitor.
**Detecting problems**: Noticing that events may be taking an unexpected direction	You know, I can tell you when I eat and when I don't eat and I can tell you when I take insulin just from looking at the chart.
**Deciding**: Involves questioning the appropriateness of standard courses of action or default decisions, considering trade-offs in ongoing plan trajectories, and sacrificing previous decisions or commitments	You know, and if somebody, if somebody's really into trying to control their diabetes. And they're doing the work of. Recording their meals and their water intake and all that, you know it's it's. What we do to help ourselves.
**Coordinating**: Managing interdependencies of activity and communication across individuals acting in roles that have common, overlapping, or interacting (and possibly conflicting) goals	No examples in data

**Table 4:** Codebook for Comments on Elements of the Integrated Report as Positive, Negative, and Suggestion.

Category	Example
**Positive**: Comment with a positive valence or expresses appreciation	It's well laid out. It's all the information you need. I mean, there's not much more.
**Negative**: Comment with a negative valence or expresses concern	If I was 70, with 70-year-old eyes, it might be a little hard to read, I don't know if it would be a little difficult for them.
**Suggestion**: A comment recommending how to modify or augment the report	Conclusions would be helpful, a slide with a little bit of conclusion, saying you're not getting enough sleep. You're not getting, you're not walking enough. You're eating too much of this. Maybe cut back on sugar. You know, just a little bit of summary of some kind of conclusion, so that they don't have to make all the conclusions themself

**Table 5:** Example of steps in iterative coding analysis of how the report is useful.

Step in Analysis	Example
Parsed transcript from participant	So it merges umm your meals, your medications and your steps and your sleep all in one place. So you can kind of get an idea of what is causing your glucose levels to change.
Investigator 1 restates parsed transcript	Supports knowing how your choices impact glucose levels
Investigator 2 restates parsed transcript	Comprehensive data in one place to explain glucose level changes
Reconciled restatement (two investigators)	The report helps me to understand how changes in my behavior explain changes in glucose levels
Investigator 1 categorizes as macrocognition function	Sensemaking: Impact of changes on interacting variables
Reconciled statement (three investigators)	The comprehensive report helps me to understand how various choices in my behavior explain changes in glucose levels
Investigator 1 groups together related statements identified by study participant and gives a group label	Understand: helps me to understand how various choices in my behavior explain changes in glucose levels (4) helps me make better decisions about my health (7) helps me in a variety of ways to self-manage my glucose level to improve my health (7) recording data for the report helps me make healthy choices when eating and drinking water (7) shows my daily activities in the sequence I do them, which helps me to understand it (11)

**Table 6:** Findings from coded interview transcripts regarding IBMS approach, clinical domain, and macrocognition framework.

IBMS	PGHD Domain	Macrocognition
Information (93)	Control (50)	Sensemaking (16)
Behavior (26)	Diet (25)	*Re*-planning (2)
Motivation (12)	Exercise (20)	Deciding (2)
	Medicine (17)	Detecting problem (1)
	Sleep (13)	Coordinating (0)
	Mood (7)	

**Table 7:** Positive and negative comments and suggestions for overall integrated report concept.

Overall Report	Positive	Negative	Suggestion
Overall report	16	1	1
Overall iconography	4	1	1
Overall layout and design	7	1	1
Tips section	0	0	2
Self-report and surveys	0	4	5
Color coding	7	0	3

**Table 8:** Positive and negative comments and suggestions for 7-day integrated report.

7-day Report Content	Positive	Negative	Suggestion
CGM Graph	2	1	0
Average daily glucose	1	0	1
Medication	2	0	0
Daily steps	1	0	0
Daily physical activity	3	2	0
Daily sleep	2	2	1
Mood	0	4	5
7-day averages table	0	1	1

CGM = Continuous Glucose Monitor.

**Table 9:** Positive and negative comments and suggestions for 3-day integrated report.

Category	3-Day Report Element	Positive	Negative	Suggestion
Graph	Sleep plot bar graph	3	3	2
Overlay	CGM graph overlay	0	1	1
Overlay	Steps chart overlay	0	2	1
Icon	Meal plot icons	1	1	0
Icon	Medication plot icons	2	1	1
Icon	Physical activity plot icons	1	0	1
Icon	Steps icon	1	2	0

**Table 10:** Categories and explanations for usefulness of the integrated report.

Category	Explanations of Value for the Report (Participant Number)
Access	provides glucose values that I would not otherwise have (3) the data comes directly to me rather than through my clinic (7) would be even more useful if it could be modified to let me manipulate the format to get more details when I want (7) shows me my own data (10) shows sleep data (10) could be more useful if it added a plot of the menstrual cycle to see how it relates to glucose levels (11)
Understand	helps me to understand how various choices in my behavior explain changes in glucose levels (4) helps me make better decisions about my health (7) helps me in a variety of ways to self-manage my glucose level to improve my health (7) recording data for the report helps me make healthy choices when eating and drinking water (7) shows my daily activities in the sequence I do them, which helps me to understand it (11)
See patterns	shows me when I ate and took insulin (7) tells me in detail things like how active I am at what time of the day (9) report makes me look back at what I ate and my activity, as well as what I forgot to report (9)
Snapshot	comprehensive report (4) gives me a good snapshot (7) gives me a snapshot for what I have done over the past seven days (9)
Questions	lets me answer questions easily (2) the detail convinces me that this is a legitimate report that lets me study how to improve my management of glucose in detail (9) helps me ask informed questions (10) helps me to ask my physician about problems to see if everyone has the same problem as me with sleep (10)
Graph	lays out how diet and exercise impact glucose (1) provides graphs (6)
Requires clinical knowledge	needs help to be understood (2) provides information that needs to be interpreted by somebody with expertise (2) might need someone to interpret due to complexity (6)
Usability	in an easy-to-understand format (1)
Remind goals	keep me accountable to previously set goals for eating, walking, etc. (9)
Draws attention	draws my attention to highlighted information by using colors (5)
Learn	categorizes types of actions using color coding that I can take to improve my health (9)
